# Activating KIRs and NKG2C in Viral Infections: Toward NK Cell Memory?

**DOI:** 10.3389/fimmu.2015.00573

**Published:** 2015-11-09

**Authors:** Mariella Della Chiesa, Simona Sivori, Simona Carlomagno, Lorenzo Moretta, Alessandro Moretta

**Affiliations:** ^1^Dipartimento di Medicina Sperimentale and Centro di Eccellenza per la Ricerca Biomedica, Università di Genova, Genoa, Italy; ^2^Dipartimento di Immunologia, IRCCS Ospedale Bambin Gesù, Roma, Italy

**Keywords:** human NK cells, activating KIRs, NKG2C, memory, HIV, HCMV

## Abstract

Natural killer (NK) cells are important players in the immune defense against viral infections. The contribution of activating killer immunoglobulin-like receptors (KIRs) and CD94/NKG2C in regulating anti-viral responses has recently emerged. Thus, in the hematopoietic stem cell transplantation setting, the presence of donor activating KIRs (aKIRs) may protect against viral infections, while in HIV-infected individuals, KIR3DS1, in combination with HLA-Bw4-I80, results in reduction of viral progression. Since, studies have been performed mainly at the genetic or transcriptional level, the effective size, the function, and the “licensing” status of NK cells expressing aKIRs, as well as the nature of their viral ligands, require further investigation. Certain viral infections, mainly due to *Human cytomegalovirus* (HCMV), can deeply influence the NK cell development and function by inducing a marked expansion of mature NKG2C^+^ NK cells expressing self-activating KIRs. This suggests that NKG2C and/or aKIRs are involved in the selective proliferation of this subset. The persistent, HCMV-induced, imprinting suggests that NK cells may display unexpected adaptive immune traits. The role of aKIRs and NKG2C in regulating NK cell responses and promoting a memory-like response to certain viruses is discussed.

## Introduction

Natural killer (NK) cells are components of the innate immune system that function as key players in anti-viral and anti-tumor immune responses. They are able to kill transformed cells with compromised HLA class I expression (“missing self hypothesis”) (1), but they can also modulate innate and adaptive immune responses by secreting chemokines/cytokines and by selecting efficient antigen-presenting cells (APCs) (2, 3). This plurality of NK cell functions is controlled by an array of inhibitory and activating receptors expressed at the cell surface. In general, in normal conditions when both types of receptors are simultaneously engaged, the inhibitory signals predominate and NK cells are prevented from killing and cytokine production (4).

The lectin-like heterodimers, CD94/NKG2A and CD94/NKG2C, and killer immunoglobulin-like receptors (KIRs) take part in the control of NK cell function.

Both CD94/NKG2A and CD94/NKG2C recognize the non-classical HLA-E molecules but their engagement results in opposite effects: NKG2A, containing an ITIM motif in its cytoplasmatic domain, transduces inhibitory signals, while NKG2C, thanks to its association with the ITAM-bearing molecule DAP-12, transduces activating signals (5).

Killer immunoglobulin-like receptors represent a family of inhibitory and activating receptors characterized by either two or three (KIR2D, KIR3D) Ig-like extracellular domains (6). Inhibitory KIRs (KIR2DL, KIR3DL) are characterized by a long cytoplasmayic tail containing ITIM motifs and bind allotypic determinants of specifically HLA class I (groups of HLA-A, -B, and -C alleles) (7). Importantly, during NK cell development, the engagement of inhibitory receptors by their self-HLA-I ligands confers intrinsic responsiveness to these cells, a property that has been referred to as “licensing” process (8–11). Activating KIRs (aKIRs) (KIR2DS, KIR3DS) are highly homologous to their inhibitory counterparts in the extracellular domain but are characterized by a short cytoplasmic tail lacking ITIMs and interact with DAP-12, a signaling polypeptide that can induce NK cell activation (12).

Activating KIR-ligands are still a matter of study. The HLA class I specificity of aKIRs has been unequivocally demonstrated only for KIR2DS1 and KIR2DS4. In particular, KIR2DS1 recognizes the C2-epitope (12–14), whereas KIR2DS4 groups C1 and C2 HLA-C alleles and HLA-A11 (15, 16). Since NK cells expressing aKIRs specific for self-HLA class I molecules could be autoreactive, tolerance has to be secured by a complementary “education” via aKIRs. In this context, NK cells expressing KIR2DS1 result hyporesponsive in HLA-C2^+^ individuals (17, 18). More recently, it has also been reported that a KIR2DS2 recombinant protein binds HLA-A11 molecule in complex with a vaccinia viral peptide (19) and that KIR2DS2^+^ NK cell clones show efficient degranulation against HLA-C1^+^ B-EBV transfected cell lines (20). aKIRs could also recognize non-HLA class I ligands. In this regard, KIR2DS4 has been shown to interact with a protein expressed on MHC class I-negative melanoma cells (21).

Because of the high homology in the ectodomain, most of the available anti-KIR antibodies are cross-reactive with inhibitory and activating isoforms, thus hampering a precise phenotypic analysis of the KIR repertoire. In recent years, however, the availability of new KIR-specific mAbs allowed to distinguish between the two isoforms (22–24).

Both the genetic polymorphisms of KIRs and their clonal expression mode contributed to generate variegated NK cell repertoires. Indeed, the human KIR gene family displays a high degree of diversity, which arises from the variability in the KIR gene content and from the allelic polymorphisms (25–27). Moreover, two main haplotypes can be identified: in addition to the complete set of inhibitory KIRs, “A” haplotypes contain a single aKIR (KIR2DS4), while “B” haplotypes have up to five aKIRs (28).

The impact of self-HLA class I molecules on the KIR repertoire is still debated and needs to be elucidated. Some investigators reported an effect of HLA-C on the KIR repertoire (29–31), suggesting an instructive model of KIR acquisition by KIR-ligands, while others described a stochastic acquisition of KIR, suggesting a random and sequential model of the repertoire (32).

## Role of Activating KIRs in Viral Infections

A growing number of studies display a significant association between the presence of aKIRs and the clinical outcome of some human diseases, including viral infections, certain tumors, and autoimmune diseases, thus suggesting that aKIRs may play a relevant role in regulating NK cell function (6, 33–36).

**Figure 1:**
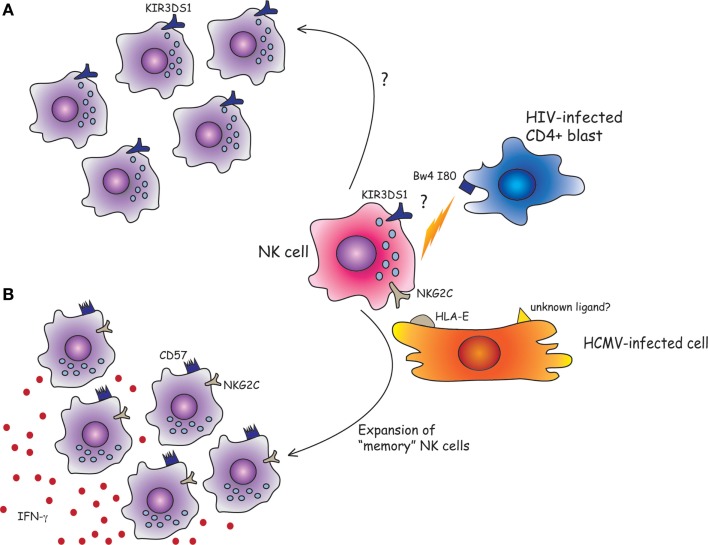
**Can human NK cells keep memory of viral infections? (A)** Although the physical interaction between KIR3DS1 and HLA-Bw4-I80 has yet to be demonstrated, it has been shown that the combined presence of KIR3DS1 gene and HLA-Bw4-I80 strongly predicts a favorable outcome for HIV-1-infected patients. Following acute HIV-1 infection, KIR3DS1^+^ NK cells might expand and efficiently control HIV infection by the killing of CD4^+^ infected blasts. **(B)** NKG2C^+^ NK cells can efficiently proliferate in response to HMCV-infected cells. The NKG2C receptor could play a crucial role in the NK cell expansion and/or maturation driven by HCMV infection by the recognition of HLA-E molecules loaded with viral peptides, or of unknown ligands expressed by HCMV-infected cells. Once exposed to a second viral challenge, the expanded “memory” long-lived NKG2C^+^ CD57^+^ NK cell subset could provide a more efficient anti-viral response (e.g., by the release of IFN-γ).

In particular, during the past few years, various studies have confirmed a role of *KIR3DS1* in HIV-1 infections (6, 37). Thus, the combined presence of *KIR3DS1 gene* and *HLA-Bw4-I80* alleles has been reported to exert a protective effect in patients with chronic HIV-1 infection. The reduction of viral load results in slow decline of CD4^+^ T cell counts and delayed progression to AIDS (37, 38). In addition, during acute HIV-1 infection, expansion of KIR3DS1^+^ NK cells (39), killing of HIV-1 infected cells, and inhibition of viral replication have been reported (40). Remarkably, this occurred only in individuals carrying *HLA-Bw4-I80* alleles. Along this line, increased *KIR3DS1* count due to copy number variants (CNVs) in *KIR3DS1/L1* locus has been associated with a lower viral set point in *HLA-Bw4-I80*^+^ individuals (41). Functional studies performed by Pelak et al. have also shown that NK cells from HLA-Bw4-I80^+^ individuals, expressing one *KIR3DS1* and two *KIR3DL1*, display large proportions of KIR3DS1^+^ NK cells in the peripheral blood and an increased resistance against HIV-1. This KIR3DS1+ NK cell expansion might represent an HIV-induced, memory-like response (Figure 1). Moreover, NK cells derived from HLA-Bw4-I80^+^ individuals with multiple copies of KIR3DS1, in the absence of KIR3DL1, were unable to mediate a robust anti-viral activity (41).

In addition to KIR3DS1, other KIRs have been associated with HIV disease progression. For example, Gaudieri et al. reported that KIR2DS2 is associated with a more rapid CD4^+^ T cell decline and progression to AIDS (42) and Soria et al. found that the functional compound genotype HLA-C1(+)/KIR2DL3(+) is associated with reduced risk of becoming an immunological non-responder to combination ART (43).

*KIR3DS1* has been also associated with a better control of H1N1 influenza A (44) but not of HTLV-1 infections (45). In addition, protective effects of aKIRs have recently been described in BK virus infection in renal transplant patients with polyoma virus-associated nephropathy (PVAN). Indeed, a significantly higher percentage of patients with BKV-associated nephropathy (BKVAN) carrying low numbers of aKIRs have been described. These findings support a role of aKIRs in the control of BKV infection after kidney transplantation (46). Moreover, *KIR2DS1, KIR3DS1*, and *KIR2DL5* would exert a protective role in the clearance of HBV. In contrast, KIR2DS2 and KIR2DS3 would favor a persistent, weak inflammatory reaction and, as a consequence, a continuous injury of liver tissues and chronic hepatitis (47).

In transplantation, various studies suggested that group B KIR haplotype is protective from viral infections. Since *Human cytomegalovirus* (HCMV) infection/reactivation is a common complication occurring after transplant in immunosuppressed subjects, many studies have focused on the possible association between aKIRs and HCMV infection. A reduced risk of HCMV reactivation has been reported in solid organ transplantation (SOT) recipients carrying more than one aKIR (haplotype B) (48). Similar results have been obtained in patients given hematopoietic stem cell transplantation (HSCT) from haplotype B donors (49). Notably, the highest protective effect has been detected in patients whose donors had a KIR genotype with more than five aKIRs or containing simultaneously *KIR2DS2* and *KIR2DS4* (50, 51). Other studies have suggested the importance of the position of aKIR genes in the telomeric region to gain a favorable effect against HCMV infection (52–54). However, all these studies analyzed KIR genotypes and/or KIR transcripts in HSCT donor/recipient pairs, but not the actual size of the NK cell subsets expressing aKIRs nor investigated whether such KIRs were functional.

Regarding the role of aKIRs in the control of certain tumors caused or at least promoted by viral infections, a protective effect of *KIR3DS1* in combination with *HLA-Bw4-I80* alleles was observed against hepatocellular carcinomas developed in chronically HCV-infected patients (55). Moreover, the presence of NK cells expressing KIR3DS1 and KIR2DS1 seems to be critical in removing human papilloma virus (HPV)-infected keratinocytes. On the other hand, the absence of *KIR3DS1* and *KIR2DS1* appears to be associated with a more frequent occurrence of respiratory papillomatosis, a rare disease caused by HPV-6/11 (56). Finally, a growing number of studies suggest a role for NK cells in the pathogenesis of autoimmune diseases. In particular, *KIR3DS1* has been associated with the development and progression of ankylosing spondylitis (57, 58).

## HCMV Infection Drives the Expansion of NKG2C^+^ and/or Activating KIRs^+^ NK Cells and may Induce Adaptive Features in NK Cells

In recent years, it has been shown that certain viral infections, mainly due to HCMV, can deeply influence NK cell development and function. HCMV infection is particularly common in human beings and usually asymptomatic in immunocompetent hosts. However, similarly to other herpes viruses, HCMV remains latent for life, undergoing occasional reactivation (59). The continuous host–HCMV interaction is probably responsible for the large degree of adaptation of NK cells to the virus. Indeed, HCMV infection promotes a persistent redistribution of the NK cell receptors repertoire, favoring a large oligoclonal expansion of NK cells with high surface expression of CD94/NKG2C and a mature self-KIR^+^NKG2A^−^ phenotypic signature (60). The imprinting induced by HCMV infection is variable among individuals and is influenced by the NKG2C gene copy number (61). It is particularly evident in case of impaired T cell immunity as in immunodeficient (62–65) or transplanted patients (HSCT or SOT) (66–68). Indeed, during the first year after transplantation, HCMV reactivation induces a rapid NK cell differentiation toward mature, fully competent CD56^dim^ NK cells expressing the NKG2C^+^ NKG2A^−^ KIR^+^ Siglec-7^−^ CD57^+^ signature. These observations, together with a previous finding that NKG2C^+^ NK cells, isolated from healthy seropositive donors, can efficiently proliferate in response to HCMV-infected fibroblasts (69), suggesting that the NKG2C receptor could play a crucial role in the NK cell expansion and/or maturation driven by HCMV infection. Along this line, Rolle et al. recently demonstrated that HLA-E expression on HCMV-infected cells is mandatory for the expansion of NKG2C^+^ NK cells (70). Since the HCMV-encoded UL40 protein stabilizes HLA-E surface expression on certain HCMV-infected targets (e.g., fibroblasts, endothelial cells), while other HCMV-derived polypeptides inhibit the surface expression of classical HLA class I molecules (71), it is possible that NKG2C^+^ NK cells receive proliferative signals from HLA-E^+^ infected cells in the absence of inhibitory KIR/HLA interactions. However, the actual role of UL40 in driving NKG2C^+^ NK cells expansion is not clear (69, 70) and it cannot be excluded that other signals provided directly or indirectly by HCMV may be involved in this process. In this context, it is worth mentioning that viral UL40 is characterized by a certain degree of polymorphism that may stimulate or inhibit NKG2C-mediated responses (72) and that HLA-E can bind different peptide sequences, e.g., those derived from Hsp60 (73, 74).

Moreover, HCMV has evolved several evasion strategies targeting NK receptors and their ligands. Indeed different HCMV-encoded proteins are capable of interfering with NK cell function (Table S1 in Supplementary Material).

On the other hand, in mice, MCMV infection unequivocally drives the expansion of NK cells expressing the activating receptor Ly49H, upon specific recognition of m157, its virus-encoded ligand (75, 76). This expanded subset displays adaptive features typical of T lymphocytes, being a long-lived cell subset capable of mounting an efficient and specific recall response (75, 77). These studies suggested that also innate cells can develop memory-like properties in response to certain pathogens, displaying adaptive cells characteristics, i.e., antigen-specificity, longevity, clonally amplified responses upon antigen (re-) exposure (Table 1).

**Table 1:** **Comparison between memory NK and T cell features**.

	Memory NK cells	Memory T cells
**General features**		
RAG-rearrangements during cell differentiation	No	Yes
Lymphoid progenitor	Yes	Yes
Requirement of γ-chain cytokines for survival/proliferation	Yes	Yes
Killing via perforin/granzymes	Yes	Yes (CD8^+^ T)
Education process to avoid autoreactivity	Yes	Yes
**ADAPTIVE FEATURES**		
Clonal expansion upon viral infection	Yes	Yes
DNA methylation at specific sites	Yes	Yes
Longevity	Yes?^a^	Yes
Ag specificity	Yes?^a^	Yes
Enhanced response to secondary challenge	Yes?^a^	Yes
MHC I/peptide recognition	Yes (through NKG2C/aKIR?)	Yes (through TCR)

*^a^These characteristics have been clearly demonstrated only for murine NK cells [Ref. (75–77)]*.

In human beings, HCMV-induced, long-living NKG2C^+^ NK cells might correspond to murine memory Ly49H^+^ NK cells, with similar memory properties. In line with this hypothesis, a recent study showed that when NKG2C^+^ NK cells were transplanted from HCMV-seropositive donors into seropositive HCST recipients, they underwent expansion and produced higher levels of IFN-γ as compared to NKG2C^+^ NK cells infused in seronegative recipients (78). This would imply that previously primed NKG2C^+^ NK cells, when exposed to a second viral challenge in the recipient elicit a more efficient anti-viral response, suggesting the acquisition of memory-like properties (78). In this context, it has been shown that NKG2C^+^ CD57^+^ NK cells, isolated from healthy HCMV-seropositive individuals, are characterized by an epigenetic remodeling at the *IFN-*γ locus. It is conceivable that this may be responsible for the enhanced IFN-γ production by NKG2C^+^ NK cells (Figure 1). This epigenetic imprinting reminds that detectable in memory CD8^+^ T cell or Th1 cells, suggesting that common molecular mechanisms may be involved in promoting the generation of memory cells. Along this line, very recent studies showed that putative adaptive/memory NK cells lack the expression of certain signaling proteins (i.e., EAT-2, Syk, and FcϵRIγ) and that these features are likely to reflect a particular DNA-methylation pattern shared by these adaptive NK cells and CTLs. This epigenetic remodeling induced by HCMV would be responsible, at least in part, for the functional specialization of adaptive NK cells that are capable of efficiently killing HCMV-infected targets via ADCC, in the presence of anti-HCMV antibodies, but show impaired response to cytokines (IL-12 and IL-18) (79–81). On the other hand, killing by ADCC can be reduced by virus-encoded FcγRs that are known as HCMV inhibitors of IgG-mediated immunity (82).

The similarities between NK and T cells described in these reports (79, 80, 83) support the unexpected concept of memory/adaptive NK cells. Indeed, with the exception of RAG-mediated gene rearrangements, NK cells share with T cells several features regarding both their development and mode of functioning. Thus, NK and T cells share a common lymphoid progenitor and undergo a “licensing” process that selects functional, non-autoreactive cells. Moreover, both cell types produce IFN-γ and TNF-α upon receptor- or cytokine-mediated activation and kill via perforin and granzymes, contained in the cytolytic granules (as in CTLs) (84, 85) (Table 1). However, while for T cells the generation of memory is a well-known process (86), at the present, neither the signals responsible for the epigenetic modifications detected in putative memory NK cells nor whether this remodeling may persist in the progeny are known (83). Further investigation focusing on the HCMV-derived ligands recognized by NKG2C will help to clarify this point.

Although NKG2C expression represents the most typical marker of NK cell expansions promoted by HCMV infection, recent reports would indicate that also aKIRs may be involved in promoting HCMV-induced NK cell differentiation (87). Thus, in patients given UCBT from donors carrying a homozygous deletion of the *NKG2C* gene, a rapid expansion of mature NK cells expressing functional aKIRs was detected (88). In the absence of NKG2C, it is possible that aKIRs may participate in the HCMV-driven NK cell maturation and in the control of infections after transplantation. This hypothesis would be in line with studies suggesting that the presence of aKIRs is protective against viral infections in different settings (18). Whether the engagement of aKIRs by (unknown) viral ligands could promote the generation of memory NK cells expressing aKIR, as hypothesized for NKG2C^+^ NK cells, is still unknown. In this context, it would also be important to investigate the level of specificity of these putative memory NK cells, expressing NKG2C and/or aKIRs, in anti-viral responses. In mice, memory Ly49H^+^ NK cells are capable of specific anti-MCMV responses while they do not respond to other pathogens (77). In human beings, an expansion of NKG2C^+^ NK cells has been reported also in individuals undergoing acute hantavirus, chikungunyia and HCV infection (89–91). However, since all these patients were HCMV seropositive, the NKG2C^+^ NK cell expansion could have been driven by a subclinical HCMV reactivation. Along this line, two recent studies showed that in HCMV-seropositive individuals undergoing acute EBV infection, NKG2C^+^ NK cells, although present in substantial proportions, did not expand, further suggesting that NKG2C^+^ NK cell expansions are HCMV specific (92, 93). On the other hand, during any anti-viral immune response, NK cells are exposed to multiple cytokines that may promote NK cell expansion and enhance IFN-γ production by NK cells through mechanisms that are not virus-specific (94).

Interestingly, a very recent study showed that antigen-specific NK cell memory could be induced in rhesus macaques after both SIV infection and vaccination (95).

Finally, it would be interesting to investigate whether memory-like NK cells expressing NKG2C and/or aKIRs may also exert efficient anti-tumor and anti-leukemia activity.

## Concluding Remarks

It is conceivable that NK cells expressing CD94/NKG2C and/or aKIRs may play a protective role in different, viral infections. This protection would be primarily based on the recognition and killing of infected cells. Although many evidences support the existence of a correlation between the presence of certain aKIR genes and protection from given viral diseases, technical difficulties in the detection of NK cell subsets expressing aKIRs together with the elusive nature of most ligands recognized by the activating NK receptors make it difficult to clearly establish their actual role. However, in a given setting such as HIV infection, the presence of KIR3DS1^+^ NK cells in association with a specific HLA-I ligand (HLA-Bw4-I80) strongly predicts a favorable outcome for infected patients.

In anti-HCMV responses, NK cells revealed unexpected adaptive features. Indeed, as discussed above, NK cells share important traits with adaptive T and B. Upon HCMV infection, NK cells may undergo clonal expansion generating long-living cells expressing NKG2C and/or aKIRs that are characterized by epigenetic modifications similar to those of memory T cells. Thus, it is possible that NK cells may develop memory responses as a strategy to keep more efficiently under control those viruses like HCMV that interact lifelong with the host, thus representing a constant challenge for the immune system. At present, we cannot exclude that also other viral infections may induce memory properties in NK cells. If the adaptive features shown by NK cells will be further substantiated and the mechanisms involved will be more precisely defined, this information may reveal useful also to implement NK cell-based treatments, such as adoptive transfer of specifically primed NK cells against given viruses.

## Conflict of Interest Statement

Alessandro Moretta is a founder and shareholder of Innate-Pharma (Marseille, France). The remaining authors declare no conflicts of interest.
